# Protocol‐Agnostic Meta Key Distribution for Encrypted Wireless Communications Enabled by Space‐Time‐Coding Metasurface

**DOI:** 10.1002/advs.202514715

**Published:** 2025-11-18

**Authors:** Xinyu Li, Long Chen, Guanxiong Shen, Kezhan Zhao, Ze Gu, Qian Ma, Jian Wei You, Tie Jun Cui

**Affiliations:** ^1^ State Key Laboratory of Millimeter Waves and Institute of Electromagnetic Space Southeast University Nanjing 210096 China; ^2^ School of Cyber Science and Engineering Southeast University Nanjing 210096 China

**Keywords:** encrypted wireless communication, physical‐layer security, programmable metasurface, secure key distribution, space‐time‐coding

## Abstract

Secure key distribution is fundamental to encrypted communications but remains challenging in indoor wireless scenarios due to high computational overhead and specialized hardware requirements. Here, a Meta Key Distribution (MKD) system based on programmable metasurface to securely distribute cryptographic keys and enable protocol‐independent encrypted communication is proposed. The metasurface embeds synchronized entropy into wireless channel by dynamically modulating the spatiotemporal properties of electromagnetic wave, thus allowing legitimate users to independently extract identical cryptographic keys. A prototype is implemented and experimentally validated in an indoor environment. The results show that the proposed MKD system achieves a key generation rate of 400 bit/s and a bit error rate below 3%, demonstrating reliable key generation performance and strong resistance to passive eavesdropping. In addition, the system can be readily integrated with standard wireless protocols such as WiFi and Bluetooth without requiring significant modifications to existing communication hardware. This metasurface‐assisted approach provides a lightweight, compatible, and secure key distribution solution, suitable for emerging applications in smart homes, the Internet of Things, and healthcare environments.

## Graphical abstract

This paper presents a programmable metasurface‐based Meta Key Distribution (MKD) system for secure, protocol‐independent key exchange in indoor wireless settings. By embedding entropy into the wireless channel, it enables lightweight, compatible cryptographic key generation. Experiments confirm reliable key agreement, low error rates, and strong eavesdropping resistance, supporting applications like IoT and smart healthcare.

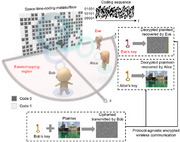

## 1 Introduction

The exponential proliferation of interconnected wireless devices has fundamentally transformed the contemporary society, facilitating the emergence of sophisticated applications in industrial automation, smart home ecosystems, healthcare technologies, and numerous other domains. ^[^ ^1^, ^2^, ^3^ ^]^ The Internet of Things (IoT) devices routinely handle sensitive personal and operational information, rendering them particularly vulnerable to diverse cybersecurity threats, including eavesdropping, man‐in‐the‐middle attacks, identity spoofing, and unauthorized data interception. ^[^ ^4^, ^5^, ^6^ ^]^ Consequently, ensuring the confidentiality, integrity, and authenticity of communications among these IoT devices is of critical importance.^[^ ^7^, ^8^, ^9^ ^]^ Contemporary IoT security frameworks rely heavily on the cryptographic techniques, including symmetric and asymmetric encryption algorithms, digital signatures, and message authentication codes, which form the cornerstone of information security.^[^ ^10^, ^11^, ^12^ ^]^ However, the security of these cryptographic systems depends critically on the secure distribution and management of cryptographic keys.^[^ ^13^ ^]^ This dependency represents the Achilles’ heel of modern cryptographic infrastructures. Without a trustworthy and efficient key distribution mechanism, even the most robust encryption schemes can be readily compromised.

To address this challenge, IoT manufacturers often pre‐install unique root keys within each device during the secure manufacturing process. Although this eliminates the need for complex key distribution protocols and infrastructures, it creates vulnerabilities, such as difficulties in updating compromised keys, increased risk during manufacturing, and limited adaptability to dynamic environments.^[^ ^14^, ^15^, ^16^ ^]^ Recent progress in quantum technology has given rise to a Quantum Key Distribution (QKD) solution.^[^ ^17^, ^18^, ^19^, ^20^ ^]^ QKD offers theoretically unconditional security based on the principles of quantum mechanics, enabling the detection of eavesdropping attempts and ensuring the secure key exchange. Nevertheless, QKD demands specialized quantum hardware such as entangled photon sources, optical fibers, and single‐photon detectors, which are infeasible to install on low‐cost commodity IoT devices. Such drawbacks underscore the need for a reliable, lightweight and compatible key distribution solution suitable for dynamic IoT ecosystems.

Recent advances in programmable metamaterials and metasurfaces^[^ ^21^, ^22^, ^23^, ^24^, ^25^ ^]^ illustrate significant potential to revolutionize wireless communication systems. Metasurfaces are subwavelength Electromagnetic (EM) structures capable of dynamically manipulating the physical characteristics of EM waves. In contrast to conventional materials, the programmable metasurfaces leverage digitally controlled meta‐atom to realize on‐demand wavefront shaping.^[^ ^26^, ^27^ ^]^ Further, Space‐Time‐Coding (STC) metasurfaces^[^ ^28^, ^29^ ^]^ have garnered significant attention due to their ability to jointly control the spatial distribution and spectral properties of EM waves, thereby enabling advanced functionalities beyond the capabilities of conventional static or purely spatially coded metasurfaces. These metasurfaces have demonstrated considerable promise in next‐generation wireless communication systems,^[^ ^30^, ^31^, ^32^ ^]^ particularly regarding to the security enhancement at the wireless Physical Layer (PHY).^[^ ^33^, ^34^, ^35^, ^36^, ^37^ ^]^ The capability to precisely control the spatial and spectral distributions of EM waves unlocks new opportunities of metasurfaces for the physical‐layer secure key distribution. In other words, a metasurface can encode entropy directly into the variations of EM field, rather than relying on upper‐layer protocols. Thus, multiple users can independently extract the identical cryptographic key based on their spatial position and received signal characteristics.

In this context, we propose a Meta Key Distribution (MKD) system that leverages the programmable metasurface to enable secure and efficient cryptographic key distribution in wireless environments. The metasurface dynamically manipulates the spatiotemporal characteristics of the EM field at the locations of transmitter (Alice) and receiver (Bob). By independently probing the time‐varying EM environments, Alice and Bob obtain highly correlated measurements, which are subsequently transformed into identical bit sequences that serve as symmetric cryptographic keys. The MKD system comprises of three essential modules, including the meta‐key initialization, generation and calibration. First, meta‐key initialization employs the spatial EM focusing techniques to concentrate EM energy at the locations of Alice and Bob while simultaneously assigning harmonic carriers to legitimate users. Second, the meta‐key generation applies STC sequences to induce synchronized frequency shifts, thereby serving as a shared entropy source for the key generation. Third, the meta‐key calibration compensates the discrepancies between the generated keys through information reconciliation and privacy amplification, ensuring the consistency and secrecy of legitimate users. Notably, we term the key generated by the proposed MKD system as the meta‐key to distinguish it from conventional physical‐layer secure keys. The prefix “meta‐” highlights that the key is derived from metasurface‐enabled EM wave manipulation, where key‐specific information is physically encoded onto the EM field through programmable space‐time modulation. Furthermore, we develop an MKD prototype and conduct experiments to demonstrate the practical feasibility of the proposed method. In the prototype, two legitimate users successfully generated a 256‐bit symmetric key and performed the encrypted communication over a standard WiFi connection. The experimental results validate the effectiveness of the proposed MKD system, achieving a key generation rate of 400 bit/s and a bit error rate below 3% between legitimate users. Compared with representative metasurface‐based key generation schemes, the proposed approach achieves comparable key generation efficiency and lower bit error rate, highlighting its potential for practical implementation in real‐world wireless scenarios. Furthermore, the key distribution and information encryption using the proposed MKD system operate at the bitstream level, enabling flexible integration with existing wireless technologies such as WiFi, Bluetooth, Zigbee, and UWB. These findings establish MKD as a lightweight, secure, and protocol‐agnostic solution for the cryptographic key distribution in next‐generation wireless systems, offering a promising enhancement to the existing security mechanisms at the wireless PHY.

## 2 Results

### 2.1 System Overview

**Figure 1:**
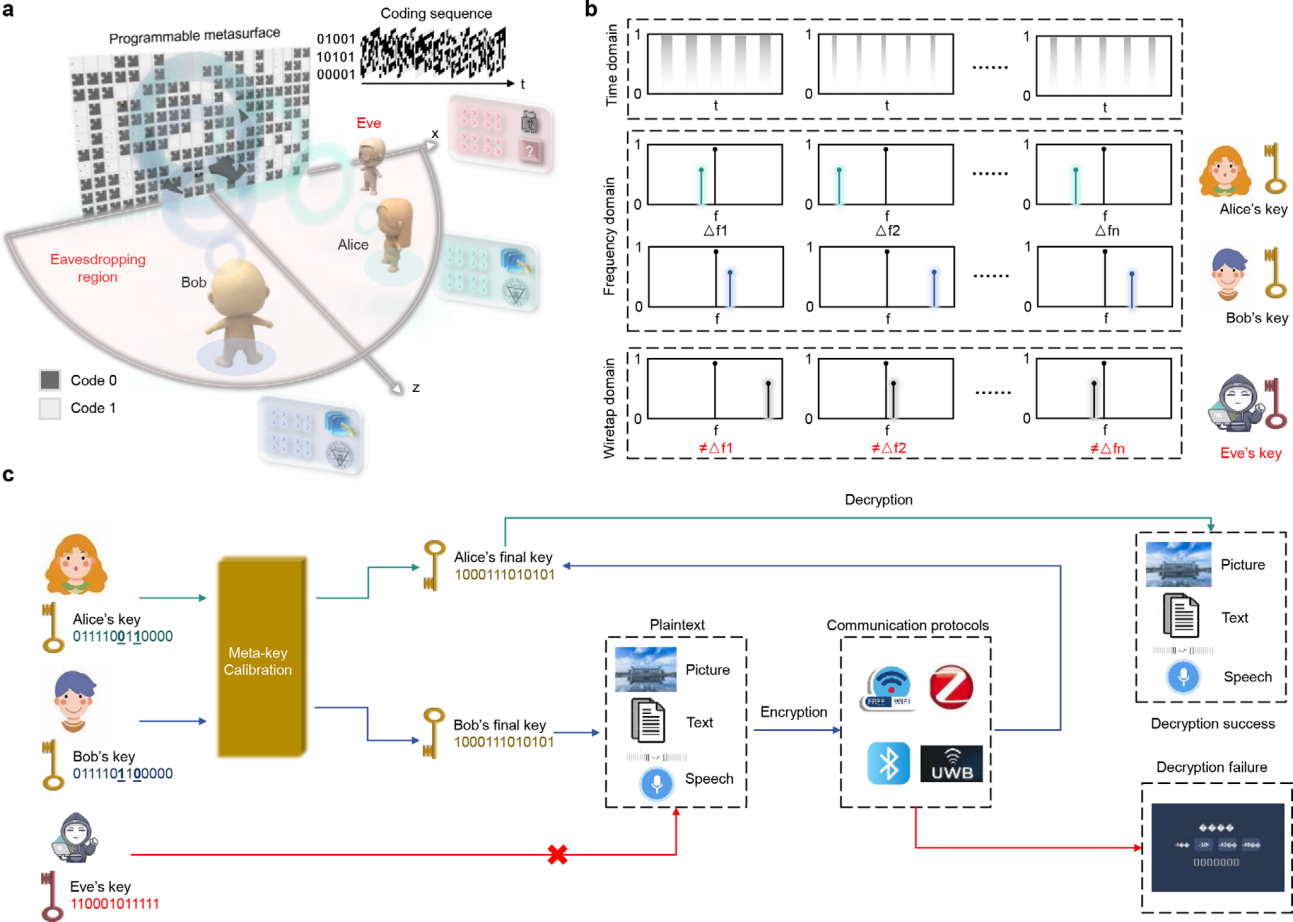
Metasurface‐empowered meta‐key distribution and encrypted wireless communications. a) The schematic diagram to distributee meta keys to the legitimate users Bob and Alice. The unauthorized user Eve in the eavesdropping region is unable to access the key‐related signals due to spatial isolation. b) Principle of MKD utilizing the STC mechanism of the programmable metasurfaces. c) Meta‐key calibration and encrypted communications using the cryptographic keys delivered by the MDK system.

As illustrated in **Figure** **1**a, the proposed MKD system consists of two legitimate communication parties, Alice and Bob, and a programmable metasurface acting as a trusted infrastructure to distribute identical cryptographic keys for symmetric encryption. The programmable metasurface periodically updates its STC patterns with random switching rate, thereby dynamically and synchronously manipulating the spectral characteristics of the EM fields that arrived at the locations of Alice and Bob. By extract the resulting variations in the EM environment, Alice and Bob independently generate identical symmetric keys based on their local measurements. Besides, the coding patterns are carefully designed to achieve precise spatial EM focusing, ensuring that the intensity of EM field remain weak in the unintended locations, i.e., the eavesdropping regions. Consequently, unauthorized users (e.g., Eve) situated in these regions are unable to detect any variations in the EM field distribution, effectively preventing over‐the‐air leakage of the cryptographic keys.

Meta‐key distribution mainly relies on the STC mechanism of programmable metasurface, as shown in Figure 1b. The metasurface is controlled by a programmable controller, such as Field Programmable Gate Array (FPGA) or Microcontroller Unit (MCU), which periodically switches the state of meta‐atoms. As the time‐modulation period T varies randomly, the harmonic frequencies assigned to Alice and Bob change accordingly in a pattern known exclusively to the metasurface, making them unpredictable to the external eavesdroppers. Subsequently, each legitimate user independently receives the incident signal in respective position and performs local frequency analysis, such as Fast Fourier Transform (FFT). The dominant frequency at that instant is then identified and quantized into binary bits, which serves as the distributed meta key. It is noted that the legitimate users Bob and Alice operate in a purely passive observation mode of the EM environment without any negotiation during the meta key distribution, thereby enhancing resistance to over‐the‐air eavesdropping on cryptographic keys.

Minor discrepancies often emerge between the meta keys generated by Alice and Bob due to noise, hardware imperfections, and environmental fluctuations. To address this issue, we incorporate a key calibration module, as illustrated in Figure 1c. After completing meta‐key generation and calibration, Alice and Bob obtain an identical binary sequence, which serves as the cryptographic key for the encrypted wireless communication. Specifically, Alice encrypts her message by performing bitwise operations with the shared meta key and transmits the resulting ciphertext using any standard wireless communication protocol. Since the encryption and decryption processes are operated directly at the bitstream level, the proposed scheme remains agnostic to specific communication protocols. Furthermore, the proposed system requires users to process the received harmonic signals using FFT. Since FFT processing is a well‐established and lightweight IP core that can be readily integrated into existing RF integrated circuits (RF‐ICs), the proposed metasurface‐enabled key distribution system remains highly promising for future deployment in IoT applications without requiring extensive hardware modifications. On the receiving end, Bob recovers the original message by decrypting the demodulated and decoded bitstream using his identical meta key. Given that both parties possess the same key, perfect message recovery is achieved. In contrast, an eavesdropper who intercepts the transmission and demodulates the bitstream cannot interpret the content due to the absence of correct key, thus ensuring the confidentiality of communication. The MKD system presents a reliable, lightweight and compatible solution for distributing the physical‐layer cryptographic keys. This system eliminates the need for designing and executing complex key exchange protocols, instead distributing the cryptographic keys through the EM manipulation capabilities of programmable metasurfaces.

### 2.2 Metasurface‐Empowered Secure Key Distribution

**Figure 2:**
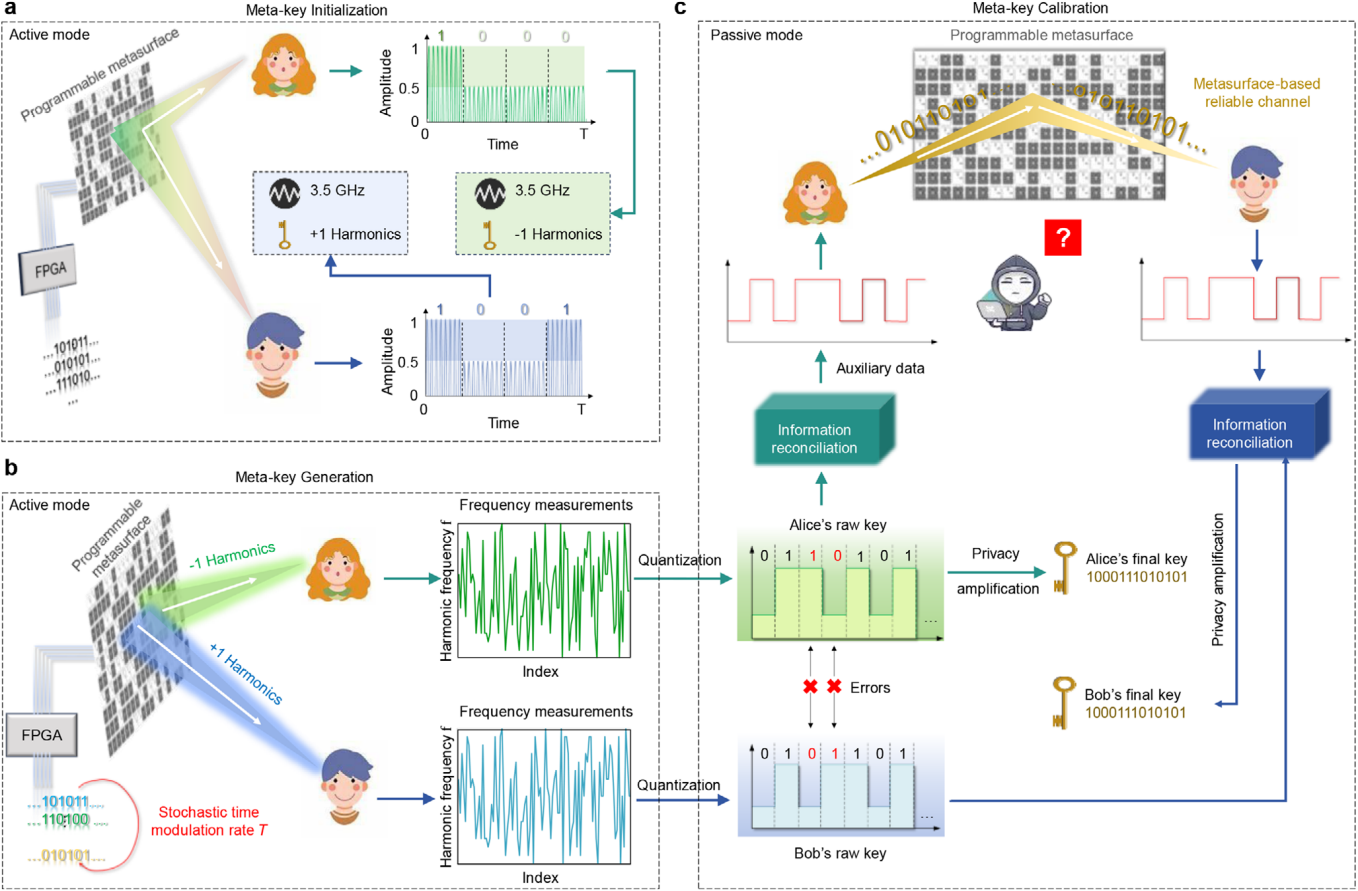
Working pipeline of meta‐key distribution, including initialization, generation and calibration. a) Meta‐key initialization, where the harmonic carriers are assigned to users via spatial EM focusing. b) Meta‐key generation, where the STC sequences induce synchronized frequency shifts that act as shared entropy sources. c) Meta‐key calibration, which calibrates the discrepancies between the generated keys via a metasurface‐based reliable channel.

Meta‐key distribution comprises of three core modules: meta‐key initialization, meta‐key generation, and meta‐key calibration. As illustrated in **Figure** **2**a, the meta‐key initialization is to securely allocate harmonic frequencies to Alice and Bob while achieving precise spatial EM focusing to prevent the potential leakage during the key distribution. The harmonic allocation process is operated through spatially‐focused binary signaling. When the programmable metasurface aims to allocate a particular harmonic to user Bob, it will modulate the EM field using predesigned space‐coding patterns that enable precise spatial EM focusing. Specifically, one coding pattern focuses the EM wave precisely at Bob's location through constructive interference, resulting in high received signal strength interpreted as bit “1”. Conversely, another coding pattern creates destructive interference at Bob's position, defocusing the energy and producing low signal levels corresponding to bit “0”. This spatial EM focusing approach enables binary signal transmissions directly to specific locations without relying on upper‐layer communication protocols. The meta‐key generation is illustrated in Figure 2b, in which the programmable metasurface dynamically modulates the reflected signal with the time modulation rate T varying and controls two harmonic signals to focus individually at the positions of Alice and Bob. In the experiments, the random sequence for time modulation rate is generated using a Cryptographically Secure Pseudo Random Number Generator (CSPRNG) in Python toolbox, which draws entropy from the operating system's randomness pool. Then, we perform the NIST SP800‐22 statistical test suite to verify the security of the generated time modulation sequence. The cryptographically secure randomness generated by the CSPRNG maintains the unpredictability of the modulation rate, thereby hindering adversaries from inferring or replicating the time‐modulation pattern. More statistical analysis to assess the quality of the generated time modulation sequence is provided in Note S1 (Supporting Information). The legitimate users then independently measure the instantaneous harmonic frequency shift of the received signal and perform quantization on this measurement, extracting raw key from the locally observed spectral features. An absolute value‐based quantization approach is used to transform the frequency measurements into binary representations by comparing them against predefined thresholds. More details about the quantization method are provided in Note S2 (Supporting Information).

Although the locally generated raw keys exhibit high correlation, the environmental interference and hardware imperfections inevitably introduce some discrepancies between the keys. Inspired by the QKD scheme, the meta‐key calibration module mitigates the discrepancies and establishes bit‐level agreement between the legitimate users via information reconciliation and privacy amplification, as illustrated in Figure 2c. Specifically, both users apply a Bose‐Chaudhuri‐Hocquenghem (BCH) error correction algorithm^[^ ^38^ ^]^ to reconcile the discrepancies in their locally generated raw keys. This BCH‐based reconciliation requires to exchange the auxiliary data over a public channel, such as a WiFi link. While this auxiliary data is derived solely from the structure of the error‐correcting code and does not directly expose the keys, it may inadvertently leak some information about the raw key. To counteract this, privacy amplification is applied after reconciliation to eliminate the residual information an adversary could obtain from observing the auxiliary data or side‐channel leakages. Specifically, we apply a privacy amplification scheme based on universal hashing. In our implementation, both Alice and Bob apply a cryptographically the secure hash function SHA‐256 to the reconciled key. This operation compresses the original key into a shorter, uniformly random bitstring, effectively removing any statistical bias or partial information leakage. The final key is thus information‐theoretically secure, even if some prior leakage has occurred during information reconciliation. Detailed structures of the BCH code, the selected parameters, and the decoding logic are provided in Note S3 (Supporting Information), while the hash‐based privacy amplification procedures are introduced in Note S4 (Supporting Information). Furthermore, the programmable metasurface is employed during the information reconciliation process to focus Alice's reconciliation signal toward Bob while suppressing signal leakage to unintended regions. Specifically, given the positions of Alice and Bob, the system employs the Gerchberg‐Saxton (GS) algorithm^[^ ^39^ ^]^ to optimize the space‐coding pattern, thereby focusing Alice's reconciliation signal toward Bob while suppressing signal leakage to unintended regions. This metasurface‐assisted approach enables flexible customization of the reconciliation channel, thereby offering the potential to mitigate Man‐in‐the‐Middle (MitM) attacks. Nevertheless, this strategy alone cannot guarantee comprehensive security. Future work will focus on developing more advanced mechanisms to further strengthen the system's robustness against sophisticated active attacks.

### 2.3 Experimental Results of Meta‐Key Distribution

**Figure 3:**
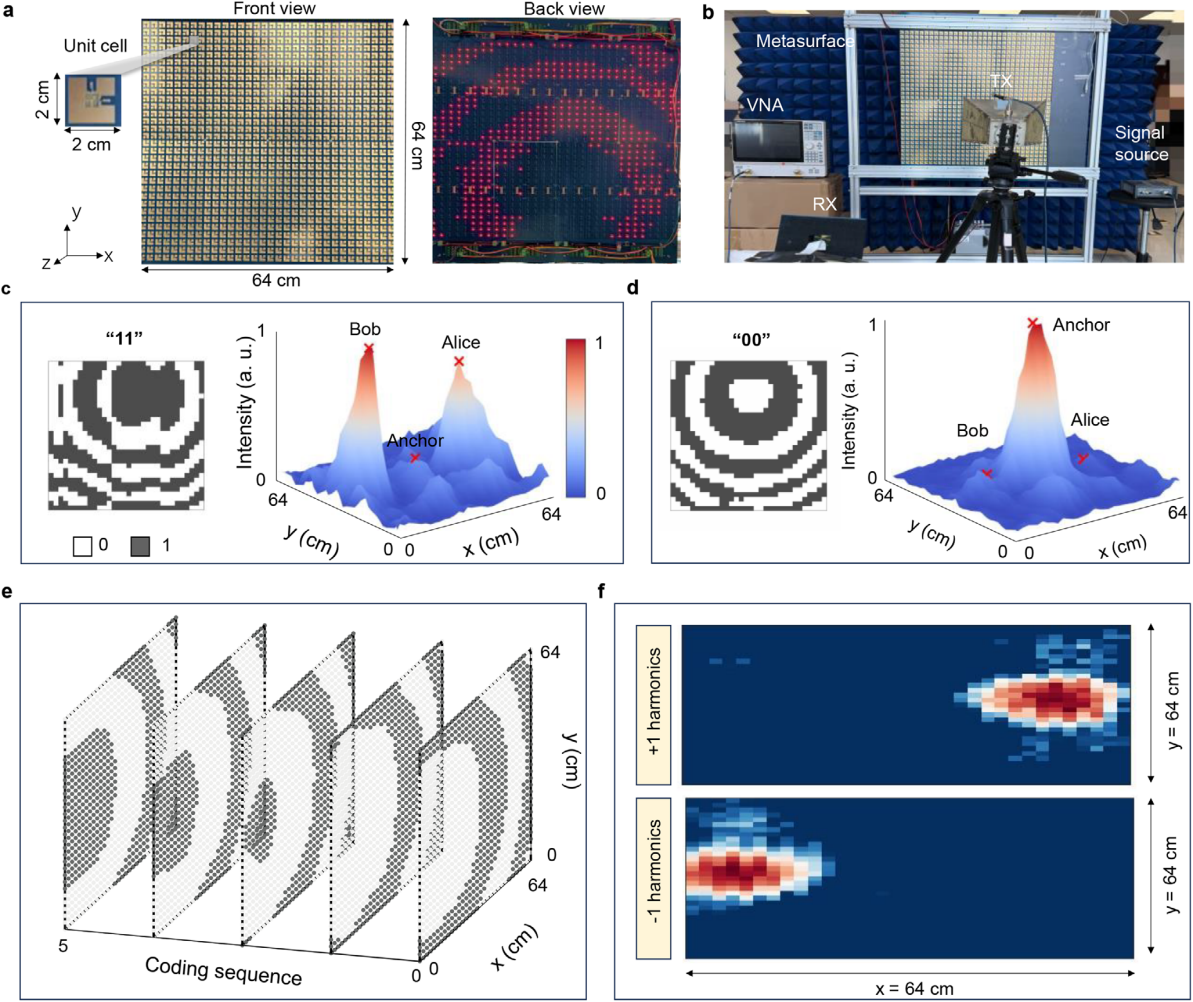
Experimental validation of the 1‐bit programmable metasurface. a) Fabricated prototype of the 1‐bit metasurface, showing its front and back layouts and unit cell. b) Measurement setup for validating the metasurface performance. c) Space‐coding pattern and the corresponding electric field distribution for the key initialization, where both Alice and Bob receive bit “1”. d) Space‐coding pattern and the corresponding electric field distribution when both Alice and Bob receive bit “0”. e) The STC sequence used by the MKD system to assign +1 and ‐1 harmonics to Alice and Bob, respectively. f) The corresponding electric field distributions of the +1 and ‐1 harmonics.

We implement a 1‐bit programmable metasurface to construct the MKD prototype system, as shown in **Figure** **3**a. To ensure compatibility with indoor communication bands while avoiding interference with existing commercial signals, we selected the relatively underutilized 3.5 GHz band for experiments. Accordingly, the metasurface is designed to operate at 3.5 GHz, enabling practical implementation of the key distribution system in realistic indoor environments. The metasurface comprises of a 32 × 32 meta‐atoms, each individually controlled by an MCU. Detailed information on the meta‐atoms is given in Note S5 (Supporting Information). The experimental setup of metasurface‐enabled key distribution is presented in Figure 3b. During the meta key initialization, the programmable metasurface utilizes four optimized space‐coding patterns to generate four two‐bit combinations: “00”, “01”, “10”, and “11”, as shown in Note S6 (Supporting Information). This design enables the users to decode their respective bits independently based solely on the signal strengths received at their positions. However, the reflected energy may be focused on a third and unintended location when both Alice and Bob are neither focused, arising a critical security challenge. In other words, the key assignment information could be leaked when an eavesdropper happens to occupy this location. To prevent this, we introduce an anchor point, which is a predefined spatial location that is regarded by the system as a trusted and physically occupied position. This point acts as an energy absorption site, where the residual EM energy is focused when neither Bob nor Alice is being targeted. Through the controlled energy management approach, we can substantially reduce the probability of key‐associated spectral signatures to reach unauthorized entities, thereby enhancing the overall security. Figure 3c shows the coding configuration and measured field distribution when Bob and Alice receive “1” simultaneously, while Figure 3d illustrates the case when they receive “0”. The optimized STC sequence and measured EM field distributions of the +1 and ‐1 harmonics are presented in Figure 3e,f. Taking the +1 harmonic as an example, its value varies dynamically with the changes of time modulation rate *T*, resulting in a frequency sequence denoted as [𝑓_1_, 𝑓_2_, …, 𝑓_𝑛_]. Meanwhile, both Alice and Bob are informed of the fundamental frequency 𝑓_0_ of the reflected EM wave in the meta key initialization module. Therefore, by subtracting 𝑓_0_ from the observed harmonic frequencies, each user can acquire a sequence of frequency shifts [𝑓_1_‐ 𝑓_0_, 𝑓_2_‐ 𝑓_0_, …, 𝑓_n_‐ 𝑓_0_], which serves as the raw entropy source for the key generation. In the proposed MKD system, key distribution is realized through dynamic modulation of the reflected signal's energy across different harmonic frequencies by the metasurface. Hence, the system holds promise for applications in static IoT scenarios, functioning in a way comparable to power‐tuning‐based approaches.

**Figure 4:**
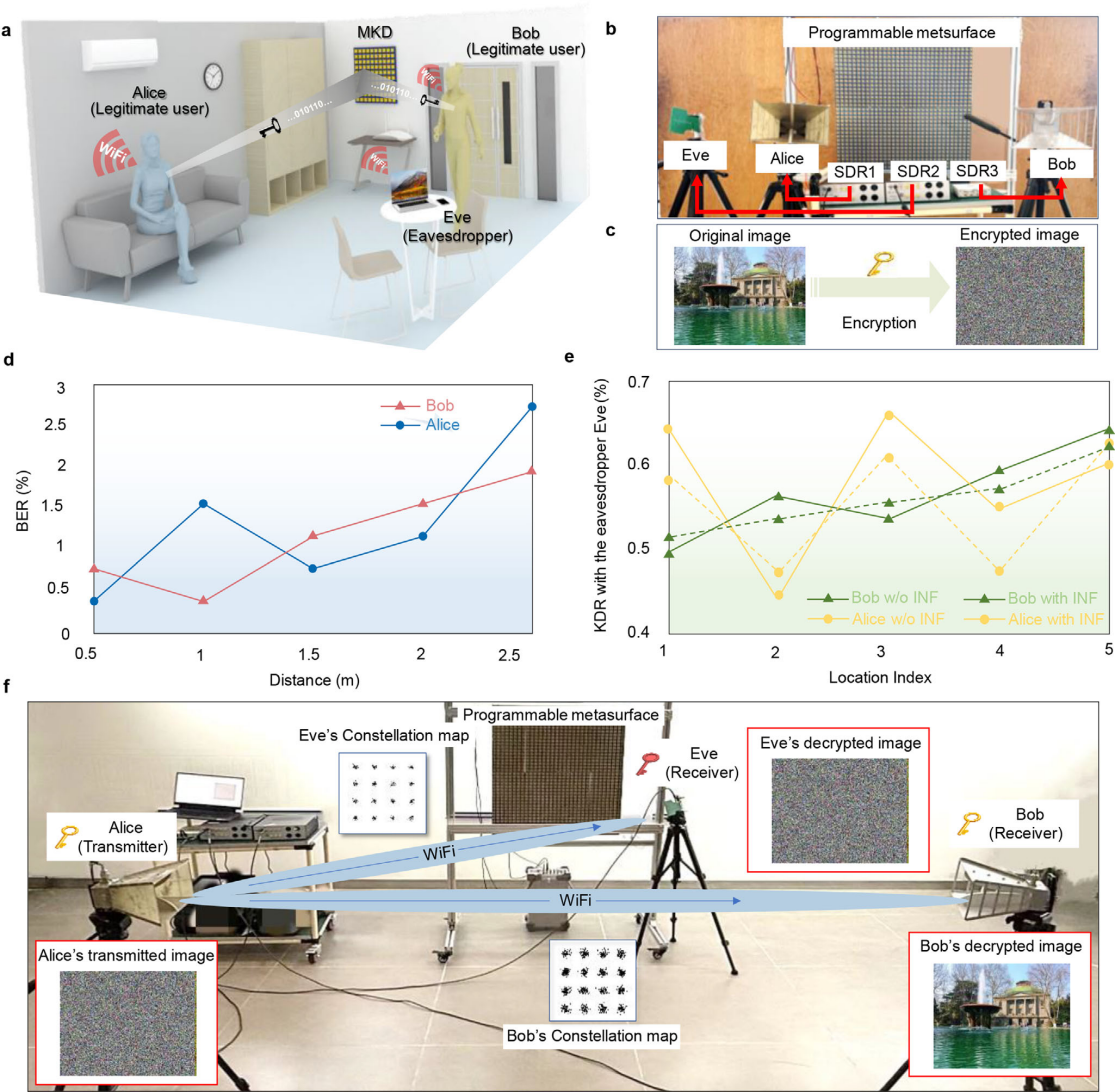
Experimental implementation of meta‐key distribution and the corresponding encrypted wireless communications. a) Schematic illustration of the encrypted communication using the meta key in an indoor environment. b) Experimental setup of the MKD system for secure key distribution. c) The original image (left) and its encrypted version (right) to be transmitted via WiFi. d) Bit error rate of the legitimate users’ keys as the distance between the programmable metasurface and users varies. e) Bit disagreement rate between the keys of the legitimate users and the eavesdropper (Eve) under different environmental interference conditions. INF denotes interference, representing a person walking within the scenario. f) WiFi‐based encrypted communications among Alice, Bob, and Eve. The legitimate user Alice encrypts the original image and transmits the encrypted version over the air. Bob and Eve receive the encrypted image via public WiFi channels. Bob successfully recovers the original image using the legitimate meta key, whereas the eavesdropper Eve fails to decrypt the ciphertext.

We develop an MKD prototype system to enable the meta‐key distribution and encrypted wireless communication. As illustrated in **Figure** **4**a, the experimental setup involves two legitimate users, Alice and Bob, who are the intended recipients of meta keys and can establish a commercial WiFi link for encrypted communications. Additionally, we introduce an eavesdropper Eve to this scenario, who attempts to intercept the distributed keys. The system aims to distribute identical cryptographic keys to Alice and Bob, while preventing any information leakage to Eve. The detailed implementation of MKD is depicted in Figure 4b. The programmable metasurface is illuminated by a 3.5 GHz single‐tone signal, which serves as the excitation carrier for meta‐key distribution. The reflected EM wave is dynamically modulated by the metasurface to encode the spatiotemporal harmonic information, which is subsequently captured by the legitimate receivers positioned at Alice and Bob's locations. Each receiver incorporates a Software‐Defined Radio (SDR) platform for signal acquisition and processing. The resulting sequence of detected frequencies then serves as the raw entropy source for cryptographic key generation. The distributed meta keys are subsequently employed for WiFi‐based encrypted wireless communications between Bob and Alice. Specifically, Alice encrypts the original image displayed in Figure 4c using the meta key, and then transmits the encrypted image in Figure 4c via a commercial WiFi link. All devices in the room, including Eve's laptop, are capable of receiving the broadcast WiFi packets, but only the users possess the correct meta key and decrypt the original content correctly. In the experiments, the programmable metasurface is powered by a 3.3 V Direct Current (DC) supply with an input current of 1 A, resulting in a total power consumption of ≈3.3 W. Considering the number of meta‐atoms and real‐time reconfiguration capability, the proposed metasurface demonstrates relatively low power consumption, making it suitable for IoT‐oriented secure communication applications. Besides, an SDR is employed as the signal source to generate a 3.5 GHz single‐tone excitation in the metasurface‐enabled key distribution system, with a power consumption of ≈120 W. Therefore, the total power consumption of the system is ≈123.3 W. In future work, a lower‐power signal source can be employed to further reduce the overall power consumption of the system.

In the proposed MKD prototype, each STC sequence is repeated multiple times on the metasurface to ensure distinct harmonic components in the reflected EM waves. To achieve this, each STC sequence is empirically configured with a duration of 25 ms in the experiments, which corresponds to a switching rate of 40 Hz. Under this configuration, the STC sequences with different time‐modulation rates can be continually repeated for more than 20 cycles, guaranteeing the generation of distinct harmonic components for accurate key extraction. Then, the harmonic frequency shift is quantized into a 10‐bit binary stream at the receiver to generate the raw key, achieving a Key Generation Rate (KGR) of 400 bit/s. Details of the frequency quantization process are provided in Note S2 (Supporting Information). The KGR achieved by the proposed MKD prototype is sufficient for typical IoT applications, such as sensor networks or smart home devices, which generally require secure key generation rates in the order of hundreds of bits per second to a few kilobits per second. However, it is lower than the key rate for high‐bandwidth applications, such as real‐time video streaming. Meanwhile, the above analysis reveals that the KGR of the proposed system is directly determined by the modulation speed of the programmable metasurface. In other words, increasing the modulation speed leads to a higher KGR. To achieve this, we will further improve the switching speed of the meta‐atom states by replacing the MCU with an FPGA and optimizing the control module of the metasurface system. In contrast, increasing the metasurface size does not directly improve KGR. Instead, it increases the complexity of controlling a large number of meta‐atoms, placing higher demands on the control module. Meanwhile, the distance between the users and the metasurface does not directly affect the key generation speed of the metasurface. Instead, signal attenuation may hinder the accurate reception of harmonic components as the distance of the metasurface and legitimate users increases, thereby potentially degrading the reliability of key extraction. To further investigate how distance affects the performance of the proposed MKD system, we analyze the Bit Error Rate (BER) of the meta‐keys received by Alice and Bob with varying distances between the programmable metasurface and user. The experimental results are illustrated in Figure 4d. The results indicate that BER increases with distance, primarily due to the diminished EM focusing capability of the metasurface over longer distances, where the target harmonics become more vulnerable to interference from other frequency components. Nevertheless, even at the maximum tested distance, Bob and Alice consistently achieve a BER below 3%, highlighting the robustness and reliability of the proposed MKD system. More importantly, BERs of Alice and Bob remain closely matched across all distances, indicating high key consistency between legitimate users. To evaluate the security and robustness of the system, we select several locations for the eavesdropper (Eve) and analyze the Bit Disagreement Rate (BDR) between Eve and the legitimate users’ keys in these scenarios. The BDR quantifies the proportion of mismatched bits between the key sequences of legitimate users and an eavesdropper, serving as an indicator of key confidentiality. A higher BDR reflects weaker correlation and thus stronger resistance to eavesdropping in physical‐layer key distribution systems. Figure 4e presents BDR results for five different eavesdropping positions. It can be observed that the BDR at all locations exceeds 40%, indicating that the eavesdropper is unable to effectively infer the legitimate keys. Furthermore, the system's performance is evaluated in a dynamic environment with moving scatterers (e.g., a person walking), as illustrated by the dashed lines in Figure 4e. The results show that the BDR between the legitimate users and Eve varies slightly compared with the baseline condition (i.e., w/o INF), which is benefit from the EM wave focusing capability of the programmable metasurface. Overall, the results in Figure 4e demonstrate that the proposed MKD system sustains stable security performance under dynamic environmental conditions. More details about the BDR results are provided in Note S7 (Supporting Information). Additionally, we compare the performance of the proposed MKD system with several state‐of‐the‐art metasurface‐based key generation approaches,^[^ ^40^, ^41^, ^42^ ^]^ as illustrated in Note S8 (Supporting Information). The comparison results indicate that the proposed MKD system achieves a competitive KGR while exhibiting strong resilience against passive eavesdropping, underscoring its effectiveness and promise for secure wireless communication applications.

Finally, the meta keys distributed by the MKD system are used for WiFi‐based encrypted wireless communication between Bob and Alice, as shown in Figure 4f. Specifically, Alice transmits the encrypted image, which appears completely randomized, over a public WiFi channel. As a result, the WiFi packets containing the ciphertext are accessible to all WiFi‐enabled devices within range. Notably, despite being closer to Alice and enjoying higher‐quality wireless link, as evidenced by a cleaner constellation diagram, Eve is unable to decrypt the received ciphertext due to the absence of the correct meta key. In contrast, Bob experiences more severe channel fading, but successfully receives the ciphertext and accurately reconstructs the original image using his locally derived meta key. This experiment validates the MKD system's effectiveness for secure key distribution and encrypted communications in real‐world wireless environment. Moreover, it inherently provides strong physical‐layer security, rendering it highly resilient to passive eavesdropping even under the challenging wireless conditions.

## 3 Conclusion

We proposed a metasurface‐empowered MKD system that embeds key‐related entropy into the physical‐layer EM environment. The system comprises of three core modules: meta‐key initialization that spatially assigns harmonic carriers to legitimate users; meta‐key generation that produces synchronized frequency sequences through spatiotemporal EM modulation; and meta‐key calibration that corrects the errors in the raw meta‐keys independently derived by different legitimate users and produces identical keys. Through experimental validation in indoor wireless environment, we demonstrated that the proposed MKD system enabled two users to reliably generate identical 256‐bit cryptographic keys without requiring interactive exchange. The generated keys were successfully applied for encrypted wireless communications in the presence of an eavesdropper, thereby confirming both reliability and spatial confidentiality of the MKD system. The MKD system offers several advantages that distinguish it from both classical and quantum key distribution techniques. Specifically, it provides a reliable, lightweight, and compatible solution for secure communications in indoor short‐range scenarios, where conventional QKD systems prove impractical due to their complexity, cost, and stringent hardware requirements. Therefore, the proposed scheme is particularly well‐suited for emerging applications in smart homes, IoT networks, and healthcare environments, where the reliable encryption is essential yet infrastructure constraints remain tight. Another strength of the MKD system lies in its protocol‐agnostic nature. The proposed MKD system is capable of integrating with existing wireless technologies such as WiFi, Bluetooth, Zigbee, and UWB without requiring extensive hardware modifications. Furthermore, the proposed MKD system shows potential resilience against Multiple‐Input Multiple‐Output (MIMO) eavesdroppers. Specifically, the EM wave focusing is realized through wave interference, where the metasurface introduces controlled phase shifts to direct energy toward a precise 3D focal region. This mechanism ensures high spatial selectivity, as the signal power rapidly decays outside the focal zone. Consequently, even if a MIMO eavesdropper is located nearby, the received signals remain significantly weaker and exhibit distinct phase and frequency characteristics from those of the legitimate users. In addition, the time‐varying coding patterns of the metasurface introduce strong spatial and temporal decorrelation, causing the channel responses at positions separated by more than half a wavelength to become statistically independent. These features collectively reduce the spatial correlation between the eavesdropper and legitimate users, thereby limiting the eavesdropper's ability to reconstruct the key information from intercepted signals.

On the other hand, the proposed MKD system still has several limitations that require further exploration. First, the current MKD system adopts a simplified threat model involving a single passive eavesdropper. The system's resistance to active jamming attacks is not explored in this work. As a result, intentional interference could potentially disrupt the key distribution process when the metasurface transmits harmonic signals containing key information. Future studies will extend the threat model to include more sophisticated adversaries and active attack scenarios, thereby improving the overall robustness and security of the MKD system. Moreover, the present prototype has not considered moving users. To overcome this limitation, real‐time user tracking can be incorporated prior to key distribution to continuously estimate the positions of legitimate users. The metasurface coding patterns can then be adaptively optimized based on the tracking results to focus EM energy on the updated user positions, maintaining stable channels and reliable key distribution even in dynamic environments. Meanwhile, the current MKD implementation operates under Line‐of‐Sight (LoS) conditions. For Non‐Line‐of‐Sight (NLoS) scenarios, future research will explore multi‐metasurface configurations, where multiple metasurfaces act as relays to cooperatively focus EM waves. This multi‐metasurface strategy has the potential to enable secure key distribution in more complex and obstructed propagation environments, further broadening the applicability of the proposed approach. Finally, the EM waves carrying key information are focused onto the positions of legitimate users through metasurface control. As shown in Figure 3f, the spatial resolution of the STC metasurface is ≈30 cm. This implies that when an eavesdropper is located within 30 cm of a legitimate user, key leakage may occur due to insufficient spatial separation. To address this potential vulnerability, future work will focus on enhancing the spatial resolution of the metasurface and reducing signal leakage to unintended locations. These improvements can be achieved by enlarging the metasurface aperture and optimizing the STC sequences to achieve more accurate EM wave focusing.

Despite these limitations, this work theoretically and experimentally demonstrates the feasibility and effectiveness of utilizing STC metasurfaces for physical‐layer key distribution. It marks an essential step toward realizing practical, low‐cost, and compatible physical‐layer security solutions for future wireless networks. Its protocol‐agnostic design, minimal hardware demands, and strong spatial discrimination align well with the evolving security needs of smart and dynamic environments. These demonstrated capabilities establish a robust groundwork for advancing the field of physical‐layer key distribution and exploring its integration into next‐generation secure communication infrastructures.

## 4 Experimental Section

### Design and Fabrication of the STC Metasurface

The programmable metasurface employed in the MKD system was fabricated using a multilayer Printed Circuit Board (PCB) process. The substrate architecture consists of a 3 mm thick F4B layer (with relative permittivity ε_r_ = 2.65, loss tangent tan δ = 0.003) bonded to a 0.5 mm thick FR4 layer (with relative permittivity ε_r_ = 4.3, loss tangent tan δ = 0.025), forming the dielectric foundation of the metasurface structure. Each unit cell was designed as a 1‐bit reconfigurable reflective element capable of binary phase modulation (0°/180°). The unit cell architecture comprised of metallic patch patterns integrated with active and passive components, including a PIN diode (SMP1345), biasing resistors, and DC blocking capacitors. Following the PCB fabrication, the diodes and passive components were manually soldered on the surface using precision assembly techniques. Detailed characterization of the reflection amplitudes and phases for individual meta‐atoms was provided in Note S5 (Supporting Information).

### Experimental Configurations

In the experimental implementation, each legitimate user was equipped with an SDR connected to a local computer to receive harmonic frequencies generated by the MKD system. Then, the meta key is extracted using FFT‐based frequency‐domain analysis and peak frequency detection. During the encrypted wireless communication experiment, Advanced Encryption Standard (AES)‐256 algorithm was employed for symmetric encryption and decryption operations. In addition to meta‐key reception, the SDR devices also serve for both modulation and demodulation of the encrypted data payloads. Specifically, the transmitter Alice encodes and modulates the ciphertext using the OpenWiFi framework^[^ ^43^ ^]^ and transmits the encrypted image via SDR; while the receivers Bob and Eve utilize the PicoScenes framework^[^ ^44^ ^]^ for WiFi signal reception, demodulation, and decoding. To ensure the compatibility with the metasurface's operational characteristics and prevent the interference with the commercial WiFi networks, the operating frequency of the encrypted wireless communication experiment was set to 3.5 GHz.

## Conflict of Interest

The authors declare no conflict of interest.

## Supporting information



Supporting Information

## Data Availability

The data that support the findings of this study are available from the corresponding author upon reasonable request.

## References

[1] J. Ma , R. Shrestha , J. Adelberg , C.‐Y. Yeh , Z. Hossain , E. Knightly , J. M. Jornet , D. M. Mittleman , Nature 2018, 563, 89. 30323288 10.1038/s41586-018-0609-x

[2] J. M. Jornet , E. W. Knightly , D. M. Mittleman , Nat. Commun. 2023, 14, 841. 36792611 10.1038/s41467-023-36621-x PMC9931692

[3] F. Zhang , Y. Guo , M. Pu , L. Chen , M. Xu , M. Liao , L. Li , X. Li , X. Ma , X. Luo , Nat. Commun. 2023, 14, 1946. 37029133 10.1038/s41467-023-37510-z PMC10081998

[4] M. A. Arfaoui , M. D. Soltani , I. Tavakkolnia , A. Ghrayeb , M. Safari , C. M. Assi , H. Haas , IEEE Commun. Surv. Tutor. 2020, 22, 1887.

[5] H. Ahmad , I. Dharmadasa , F. Ullah , M. A. Babar , ACM Comput. Surv. 2023, 55, 38.

[6] F. Dong , W. Wang , X. Li , F. Liu , S. Chen , L. Hanzo , IEEE Trans. Green Commun. Netw. 2023, 7, 537.

[7] M. Wei , H. Zhao , V. Galdi , L. Li , T. J. Cui , Nat. Electron. 2023, 6, 610.

[8] S. Venkatesh , X. Lu , B. Tang , K. Sengupta , Nat. Electron. 2021, 4, 827.

[9] O. A. Khashan , R. Ahmad , N. M. Khafajah , Ad. Hoc. Netw. 2021, 115, 102448.

[10] F. Zhang , Y. Guo , M. Pu , L. Chen , M. Xu , M. Liao , L. Li , X. Li , X. Ma , X. Luo , Nat. Commun. 2023, 14, 1946. 37029133 10.1038/s41467-023-37510-z PMC10081998

[11] Z. Yu , H. Li , W. Zhao , P.‐S. Huang , Y.‐T. Lin , J. Yao , W. Li , Q. Zhao , P. C. Wu , B. Li , P. Genevet , Q. Song , P. Lai , Nat. Commun. 2024, 15, 2607. 38521827 10.1038/s41467-024-46946-w PMC10960874

[12] G. Qu , W. Yang , Q. Song , Y. Liu , C.‐W. Qiu , J. Han , D.‐P. Tsai , S. Xiao , Nat. Commun. 2020, 11, 5484. 33127918 10.1038/s41467-020-19312-9 PMC7603497

[13] G. J. Fan‐Yuan , S. Wang , Light Sci. Appl. 2025, 14, 24. 39743622 10.1038/s41377-024-01693-x PMC11693748

[14] H. Xiong , T. Yao , H. Wang , J. Feng , S. Yu , IEEE Internet Things J. 2021, 9, 401.

[15] A. Javadpour , F. Ja'fari , T. Taleb , Y. Zhao , B. Yang , C. Benzaïd , IEEE Internet Things J. 2023, 11, 7525.

[16] M. Yang , L. Zhu , Q. Zhong , R. El‐Ganainy , P. Y. Chen , Nat. Commun. 2023, 14, 1145. 36854673 10.1038/s41467-023-36508-x PMC9974995

[17] Y. Li , W.‐Q. Cai , J.‐G. Ren , C.‐Z. Wang , M. Yang , L. Zhang , H.‐Y. Wu , L. Chang , J.‐C. Wu , B. Jin , H.‐J. Xue , X.‐J. Li , H. Liu , G.‐W. Yu , X.‐Y. Tao , T. Chen , C.‐F. Liu , W.‐B. Luo , J. Zhou , H.‐L. Yong , Y.‐H. Li , F.‐Z. Li , C. Jiang , H.‐Z. Chen , C. Wu , X.‐H. Tong , S.‐J. Xie , F. Zhou , W.‐Y. Liu , Y. Ismail , et al., Nature 2025, 640, 47. 40108471 10.1038/s41586-025-08739-z

[18] W. Li , L. Zhang , H. Tan , Y. Lu , S.‐K. Liao , J. Huang , H. Li , Z. Wang , H.‐K. Mao , B. Yan , Q. Li , Y. Liu , Q. Zhang , C.‐Z. Peng , L. You , F. Xu , J.‐W. Pan , Nat. Photonics 2023, 17, 416.

[19] W. Zhang , T. van Leent , K. Redeker , R. Garthoff , R. Schwonnek , F. Fertig , S. Eppelt , W. Rosenfeld , V. Scarani , C. C.‐W. Lim , H. Weinfurter , Nature 2022, 607, 687. 35896650 10.1038/s41586-022-04891-y PMC9329124

[20] W.‐Z. Liu , Y.‐Z. Zhang , Y.‐Z. Zhen , M.‐H. Li , Y. Liu , J. Fan , F. Xu , Q. Zhang , J.‐W. Pan , Phys. Rev. Lett. 2022, 129, 050502. 35960585 10.1103/PhysRevLett.129.050502

[21] C. Qian , I. Kaminer , H. Chen , Nat. Commun. 2025, 16, 1154. 39880838 10.1038/s41467-025-56122-3 PMC11779837

[22] D. X. Xia , J. Q. Han , Y. J. Mu , L. Guan , X. Wang , X. J. Ma , L. H. Zhu , T. G. Lv , H. X. Liu , Y. Shi , L. Li , T. J. Cui , Nat. Commun. 2024, 15, 10358. 39609441 10.1038/s41467-024-54800-2 PMC11604785

[23] X. Qiu , J. Zhang , Y. Fan , J. Zhou , L. Chen , D. P. Tsai , Nat. Commun. 2025, 16, 2437. 40069144 10.1038/s41467-025-57715-8 PMC11897169

[24] H. Jiang , Y. Chen , W. Guo , Y. Zhang , R. Zhou , M. Gu , F. Zhong , Z. Ni , J. Lu , C.‐W. Qiu , W. Gao , Nat. Commun. 2024, 15, 8347. 39333579 10.1038/s41467-024-52632-8 PMC11436760

[25] Y. Li , C. T. Chan , E. Mazur , Light Sci. Appl. 2021, 10, 203. 34588416 10.1038/s41377-021-00642-2 PMC8481486

[26] G.‐B. Wu , J. Y. Dai , K. M. Shum , K. F. Chan , Q. Cheng , T. J. Cui , C. H. Chan , Nat. Commun. 2023, 14, 5155. 37620303 10.1038/s41467-023-40717-9 PMC10449906

[27] S. S. Kruk , L. Wang , B. Sain , Z. Dong , J. Yang , T. Zentgraf , Y. Kivshar , Nat. Photonics 2022, 16, 561.

[28] L. Zhang , M. Z. Chen , W. Tang , J. Y. Dai , L. Miao , X. Y. Zhou , S. Jin , Q. Cheng , T. J. Cui , Nat. Electron. 2021, 4, 218.

[29] G. B. Wu , J. Y. Dai , Q. Cheng , T. J. Cui , C. H. Chan , Nat. Electron. 2022, 5, 808.

[30] H. Zhao , Y. Shuang , M. Wei , T. J. Cui , P. D. Hougne , L. Li , Nat. Commun. 2020, 11, 3926. 32764638 10.1038/s41467-020-17808-y PMC7413398

[31] R. Z. Jiang , Q. Ma , Z. Gu , J. C. Liang , Q. Xiao , Q. Cheng , T. J. Cui , Adv. Sci. 2024, 11, 2306181. 10.1002/advs.202306181 PMC10870054 38064159

[32] M. Liu , P. Huo , W. Zhu , C. Zhang , S. Zhang , M. Song , S. Zhang , Q. Zhou , L. Chen , H. J. Lezec , A. Agrawal , Y. Lu , T. Xu , Nat. Commun. 2021, 12, 2230. 33850114 10.1038/s41467-021-22462-z PMC8044217

[33] Y. Zheng , K. Chen , Z. Xu , N. Zhang , J. Wang , J. Zhao , Y. Feng , Adv. Sci. 2022, 9, 2204558. 10.1002/advs.202204558 PMC9731697 36253150

[34] H. L. Wang , H. F. Ma , Y. K. Zhang , T. Y. Zhang , S. Sun , Y. T. Chen , T. J. Cui , Sci. Adv. 2024, 10, adk7557. 10.1126/sciadv.adk7557 PMC11122686 38787949

[35] P. Zheng , Q. Dai , Z. Li , Z. Ye , J. Xiong , H.‐C. Liu , G. Zheng , S. Zhang , Sci. Adv. 2021, 7, abg0363. 10.1126/sciadv.abg0363 PMC8139587 34020956

[36] Z. Li , X. Kong , J. Zhang , L. Shao , D. Zhang , J. Liu , X. Wang , W. Zhu , C.‐W. Qiu , Laser Photonics Rev. 2022, 16, 2200113.

[37] J. Tian , Z. Li , C. He , W. Zhu , Laser Photonics Rev. 2025, 19, 2500395.

[38] C. Huth , R. Guillaume , T. Strohm , P. Duplys , I. A. Samuel , T. Güneysu , Comput. Netw. 2016, 109, 84.

[39] L. Li , T. Jun Cui , W. Ji , S. Liu , J. Ding , X. Wan , Y. Bo Li , M. Jiang , C.‐W. Qiu , S. Zhang , Nat. Commun. 2017, 8, 197. 28775295 10.1038/s41467-017-00164-9 PMC5543116

[40] N. Gao , Y. Han , N. Li , S. Jin , M. Matthaiou , IEEE Wireless Commun. 2024, 31, 355.

[41] G. Li , L. Hu , P. Staat , H. Elders‐Boll , C. Zenger , C. Paar , A. Hu , IEEE Wireless Commun. 2022, 29, 146.

[42] Z. Wan , K. Huang , X. Xu , M. Yi , H.‐M. Wang , Z. Zhu , L. Jin , IEEE Internet Things J. 2024, 11, 26877.

[43] X. Jiao , W. Liu , M. Mehari , M. Aslam , I. Moerman , Proc. IEEE Veh. Technol. Conf., 2020, 1‐2.

[44] Z. Jiang , T. H. Luan , X. Ren , D. Lv , H. Hao , J. Wang , K. Zhao , W. Xi , Y. Xu , R. Li , IEEE Internet Things J 2021, 9, 4476.

