# Supplementary material for: Protocol‐Agnostic Meta Key Distribution for Encrypted Wireless Communications Enabled by Space‐Time‐Coding Metasurface
Source: Adv Sci (Weinh). 2025 Nov 18;13(7):e14715. doi: 10.1002/advs.202514715 (PMC12866815; doi:10.1002/advs.202514715)
Supplement: Supplementary file 1 — Supporting Information [file ADVS-13-e14715-s001.docx]

Supporting Information

**Protocol-agnostic key distribution enabled by space-time metasurface for encrypted wireless communications**

*Xinyu Li, Long Chen, Guanxiong Shen, Kezhan Zhao, Ze Gu, Qian Ma, Jian Wei You^*^, and Tie Jun Cui^*^*

This Supplementary Information includes:

Supplementary Note 1. Statistical Analysis of the Random Time Modulation Sequence

Supplementary Note 2. Strategy of frequency quantization for key generation

Supplementary Note 3. Information reconciliation for meta-key calibration

Supplementary Note 4. Privacy amplification for meta-key calibration

Supplementary Note 5. Design of the 1-bit programmable metasurface

Supplementary Note 6. Impact of the anchor point in reducing signal leakage

Supplementary Note 7. Bit disagreement rate with the eavesdropper located at different locations

Supplementary Note 8. Performance comparison with state-of-the-art metasurface-based key generation approaches

Supplementary References

**Supplementary Note 1. Statistical Analysis of the Random Time Modulation Sequence**

To ensure the randomness and unpredictability of the time modulation rate T, we conducted a statistical evaluation of its randomness source. In the experimental implementation, the random modulation sequence was generated using Python’s secrets.randbelow(.) function. The utilized secrets module interfaces directly with the operating system’s entropy pool and operates as a Cryptographically Secure Pseudo Random Number Generator (CSPRNG), ensuring high-entropy randomness suitable for security-critical applications. Then, each sampled value Ti∈[0, N-1] was encoded into a binary bitstream using m=⌈log2N⌉=10 bits per symbol. Meanwhile, the metasurface was controlled by a Microcontroller Unit (MCU) in our prototype, which constrained the switching frequency f_s_ of meta-atom states to approximately 4 kHz, i.e., f_s_ ∈ [0, 4000] Hz. In the experimental setup, each optimized space-time coding (STC) sequence consists of five space-coding patterns. Finally, the maximum ±1 harmonic frequency shift observed by a legitimate user is 4000 Hz/5=800 Hz. As a result, N is set to 800 when generating the random sequences of time modulation rate.

Then, we employed the NIST SP800-22 statistical test suite to assess the statistical quality of the generated sequences, which is implemented through the nistrng library in Python. All sub-tests, including Frequency, Runs, FFT, and Serial, were successfully passed with p-values greater than 0.01, indicating no detectable deviation from ideal random behavior. Furthermore, the average Shannon entropy calculated across the ten test sequences was 0.9601 bits per bit, which is close to the theoretical maximum of 1 bit per bit. These results verify the high entropy and unpredictability of the time modulation sequence produced by the CSPRNG. The inherent cryptographic quality of the entropy source guarantees that the modulation rate T remains unpredictable and uncorrelated across transmissions, effectively preventing any adversary from inferring or reproducing the temporal modulation pattern.

**Supplementary Table I. NIST Statistical Test**

| **Statistical test** | ***p*-value** |
| --- | --- |
| Frequency | 0.511 |
| Block Frequency | 0.260 |
| Runs | 0.330 |
| Longest Runs | 0.158 |
| Rank | 0.275 |
| FFT | 0.092 |
| Non Overlapping Template | 0.218 |
| Serial | 0.625 |

**Supplementary Note 2. Strategy of frequency quantization for key generation**

In our proposed system, the maximum switching rate of the metasurface codebook is 4 kHz, and the length of the space-time coding sequence is set to 5. Consequently, the maximum frequency shift for the +1/-1 harmonics is 800 Hz. The legitimate users independently measure the instantaneous harmonic frequency of the received signal and then apply quantization to these frequency measurements to derive raw keys. The quantization process is described as follows.

- The frequency range of 0-800 Hz is discretized into intervals of 10 Hz, specifically: 10, 20, ..., up to 800 Hz. This allows us to reduce the continuous frequency values into a manageable set of discrete values that can be effectively used in key generation.
- Each of these quantized frequency values is then converted into its binary representation. For example:
  - A frequency of 10 Hz is represented as the binary string “0000001010”.
  - A frequency of 800 Hz is represented as the binary string “1100100000”.
- When a user detects a harmonic frequency shift of 766 Hz, for instance, the value is rounded and falls within the interval (760, 770]. Then, the measured frequency shit is quantized to 770 Hz and represented as the binary string “1100000010”.

This quantization process ensures that the raw key generated from the spectral features is consistent and compatible with cryptographic operations in the subsequent stages of key generation and reconciliation.

**Supplementary Note 3. Information reconciliation for meta-key calibration**

Information reconciliation is a crucial process in physical-layer key generation systems to ensure that two legitimate users, who have independently derived similar but non-identical keys due to noise and hardware inconsistencies, can correct any mismatches and obtain an identical shared key. In our MKD system, we employ a classical error correction method based on the Bose-Chaudhuri-Hocquenghem (BCH) algorithm^1^ to achieve this reconciliation. The BCH code is a cyclic error-correcting code defined over a finite field, capable of detecting and correcting multiple bit errors within a fixed-length binary string. A BCH code is typically denoted as BCH(n, k, t), where n is the length of the codeword, k is the length of the original message, and t is the maximum number of correctable bit errors. The BCH-based reconciliation procedure involves the following steps:

1. Encoding (Alice): Alice encodes her raw meta-key m(x) using the BCH encoder, producing a codeword c(x) of length n, where c(x) = m(x) · g(x), and g(x) is the generator polynomial of the BCH code.
2. Syndrome generation and transmission: Alice computes the syndrome vector s based on c(x), or alternatively extracts parity-check information (e.g., redundant bits), and transmits this auxiliary information (i.e., syndrome) to Bob over a public but authenticated channel.
3. Decoding (Bob): Bob compares his own raw key c’(x), which may contain errors, against the received auxiliary information. Using BCH decoding, typically involving syndrome calculation, error location via the Berlekamp-Massey or Euclidean algorithm, and error correction, Bob reconstructs the original codeword c(x).
4. Key recovery: After decoding, Bob retrieves the original message m(x) from the corrected codeword by removing the redundancy, thus recovering a key identical to Alice’s with high probability.

The generator polynomial g(x) of the BCH code satisfies^1^:

g(x) = LCM{M₁(x), M₂(x), ..., M₂ₜ(x)} (1)

where LCM represents Least Common Multiple. Mᵢ(x) denotes the minimal polynomial of αⁱ over Galois Field of order 2, namely GF(2). t is the number of correctable errors. Supplementary Fig. 1 illustrates the overall flow of the information reconciliation process using the BCH algorithm, including encoding, transmission of auxiliary data, and error correction at the receiver.


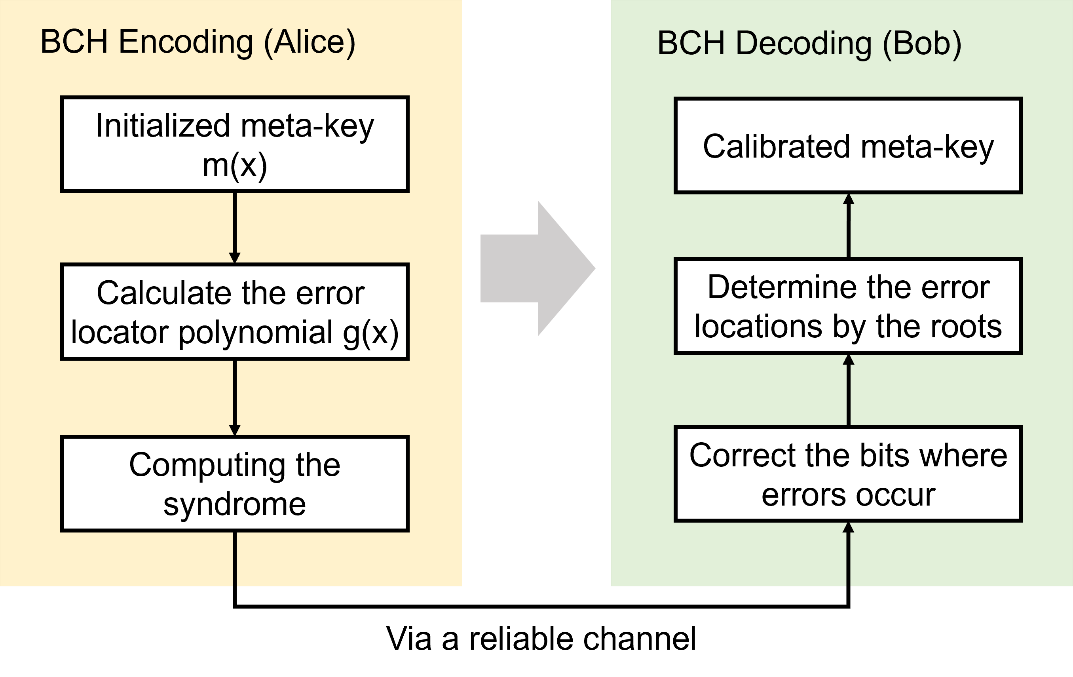


**Supplementary Fig. 1. Working pipeline of the BCH-based key calibration scheme.**

**Supplementary Note 4. Privacy amplification for meta-key calibration**

Privacy amplification is a critical post-processing step in physical-layer key generation systems, designed to eliminate any residual information that might be partially known to an eavesdropper after the key reconciliation phase. Even though the BCH-based error correction procedure aligns the raw keys of Alice and Bob, a passive adversary may still acquire partial knowledge about the key through side-channel leakage, public syndrome exchange, or noise-induced statistical correlation. To mitigate this risk and ensure information-theoretic security, we apply a privacy amplification scheme based on universal hashing. In our implementation, both Alice and Bob apply a cryptographically secure hash function (SHA-256) to the reconciled key. This function produces a fixed-length, uniformly random bitstring, effectively transforming the key into a new, shorter, and more secure form. The SHA-256 hash function is designed to eliminate any statistical bias, ensuring that the final key is uniformly distributed, regardless of any partial information leakage that may have occurred during reconciliation. The operation reduces the potential leakage from any prior steps and provides information-theoretic security, even in cases where some adversarial observations may have occurred during the key reconciliation process.

Mathematically, if the reconciled key is denoted by 𝐾 ∈ {0, 1}^n^, and eavesdropper’s maximum mutual information is bounded by ϵ, the final secure key 𝐾′∈{0,1}^m^ is derived by applying a universal hash function ℎ: {0,1}^n^→{0,1}^m^, such that

𝐾’ = h(𝐾), with m≤n−2log_2_(1/ϵ). (2)

This ensures that the adversary’s information about 𝐾’ is negligible, and the final key is statistically indistinguishable from a uniformly random string. Supplementary Fig. 2 gives a conceptual diagram of the privacy amplification process following the BCH-based information reconciliation. In summary, privacy amplification strengthens the confidentiality of the MKD system by transforming reconciled keys into provably secure cryptographic material, even in the presence of partial leakage.


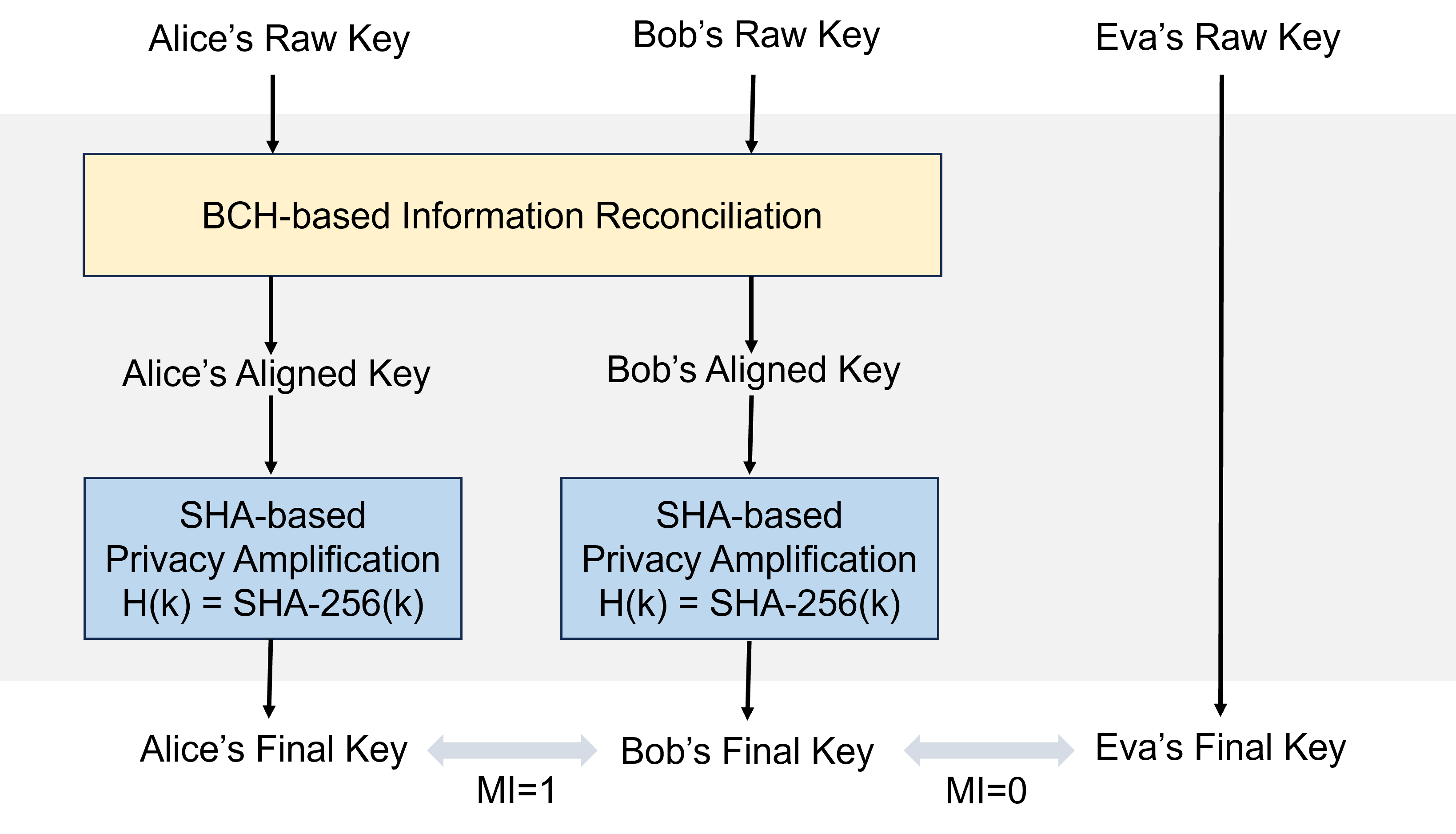


**Supplementary Fig. 2. Conceptual diagram of the SHA-based privacy amplification process following information reconciliation.**

**Supplementary Note 5. Design of the 1-bit programmable metasurface**

The metasurface used in this work was designed and fabricated using a standard multilayer printed circuit board (PCB) proces1. As displayed in Supplementary Fig. 3a, each unit cell (meta-atom) consists of a sandwich structure formed by two dielectric substrates: an upper layer of F4B material (relative permittivity of 2.65 and loss tangent of 0.003) with a thickness of 3 mm, and a bottom layer of FR4 (relative permittivity of 4.3 and loss tangent of 0.025) with a thickness of 0.5 mm. The metallic patterns on each layer were etched using photolithography and copper-clad lamination, forming a four-layer stacked architecture. Two PIN diodes are integrated into each meta-atom to achieve dynamic 1-bit phase modulation by switching between ON and OFF states with a 180° phase difference. A microstrip line is embedded to guide a portion of the incident energy to an RMS power detector, enabling simultaneous EM sensing functionality.

The fabricated metasurface array comprises of 32 × 32 elements and supports polarization-insensitive reflection control. During assembly, all passive components and active switching devices (including PIN diodes and RMS detectors) were manually soldered onto the PCB under a microscope. Electrical biasing and sensing signal routing are implemented on separate layers to minimize interference. The overall array is connected to a microcontroller unit and a power supply through a dense pin array for real-time programmability. As shown in Supplementary Fig. 3b, the S-parameters confirm that the reflection magnitude exceeds −3 dB within the operating band around 3.5 GHz, and the phase difference between states remains within 180 ± 9°, validating its suitability for dynamic beam steering and EM sensing applications.


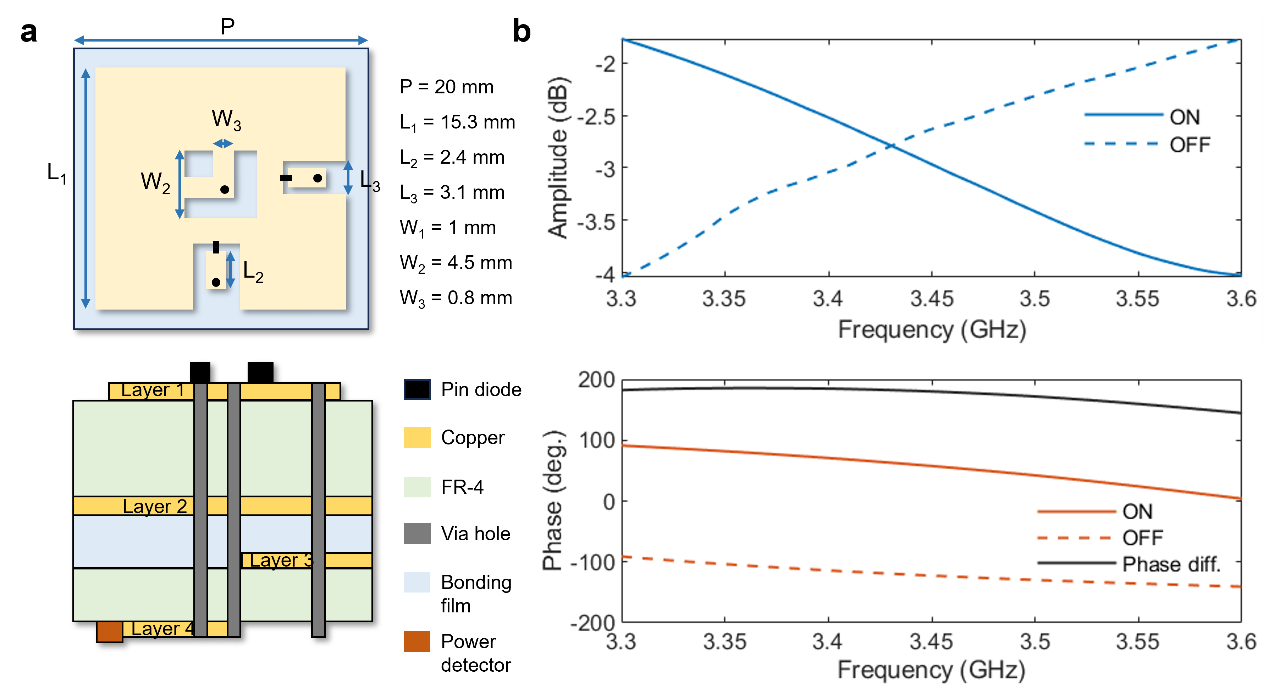


**Supplementary Fig. 3.** **Fabrication and validation of 1-bit programmable metasurface.** **a.** Overhead and side views of the meta-atom structure. **b.** Simulated reflection amplitudes and phases of the meta-atom.

**Supplementary Note 6. Impact of the anchor point in reducing signal leakage**

Supplementary Fig. 4 illustrates four spatial coding patterns employed by the fabricated metasurface during the meta-key initialization process, along with their corresponding electric field distributions. These patterns define the initial bit values received by legitimate users (Bob and Alice) and form the foundation of our physical-layer key establishment scheme. Supplementary Figs. 4e-h present the electric field distributions under the coding configuration Figs. 4a-d. These measurements confirm the metasurface’s capability to precisely control wavefront propagation and independently encode different bit combinations for the intended recipients. During the meta-key initialization phase, the metasurface in the MKD system employs the four optimized space-coding patterns corresponding to generate the following two-bit information: “00”, “01”, “10”, and “11”. For the coding patterns corresponding to “10”, “01”, and “11”, the energy is focused toward Bob, Alice, or both, respectively. In these cases, energy at other spatial positions (including those accessible to potential eavesdroppers) is minimal due to the precise electromagnetic wave focusing, thereby ensuring that no meaningful signal is leaked. However, for the “00” coding pattern, the metasurface is configured such that no energy is focused toward either Alice or Bob. In this case, residual electromagnetic energy may be unavoidably focused toward a third spatial location. Without additional protection, an eavesdropper located at this residual focal point could interpret the received bit as a “0” through straightforward bit inversion when Alice and Bob obtain the same bit “0”, thereby compromising bit-level secrecy.


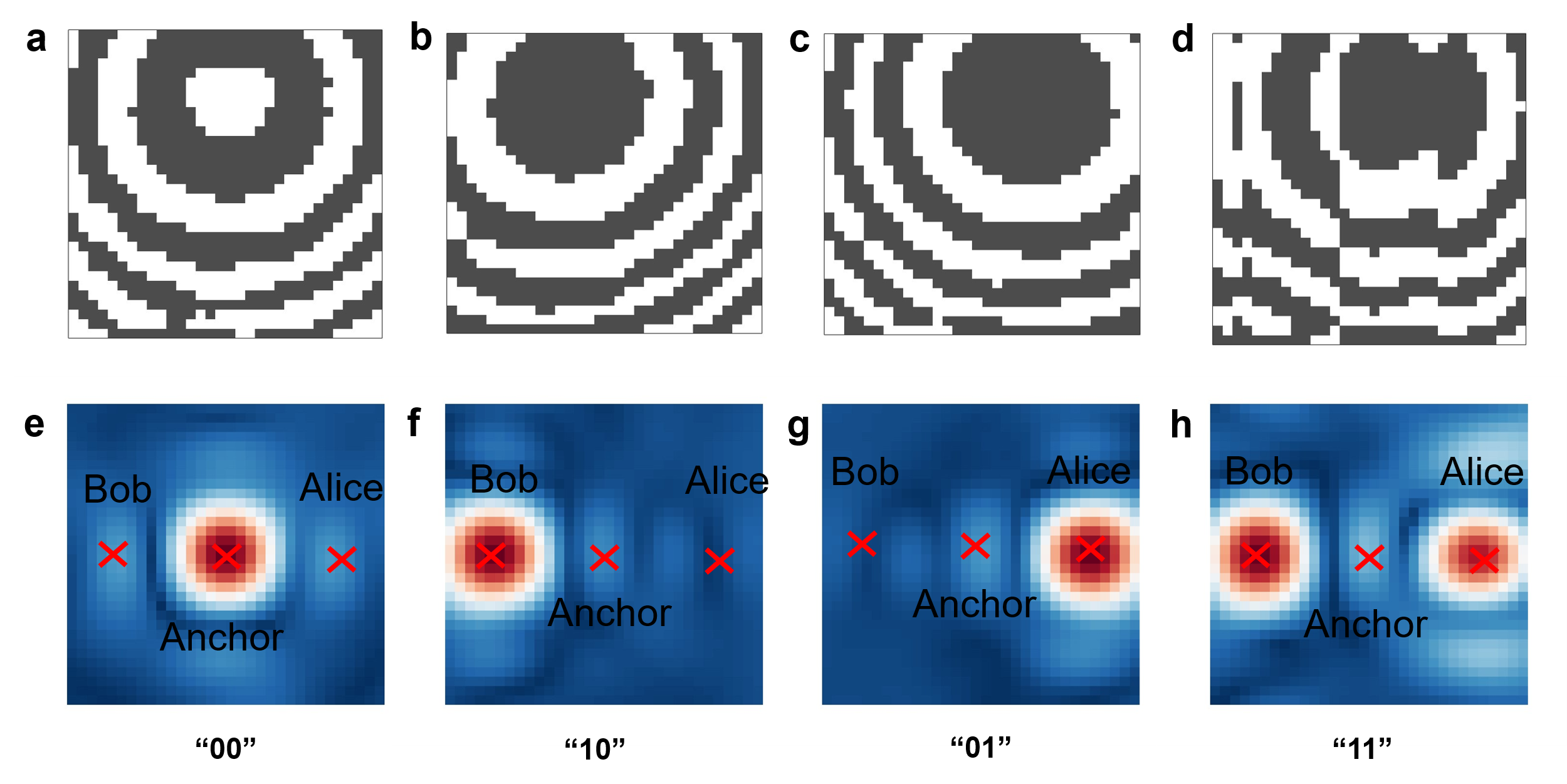


**Supplementary Fig. 4.** **Four spatial coding patterns for meta-key initialization. a** Coding pattern when both Bob and Alice receive a “0”. **b** Coding pattern when Bob receives a “1” and Alice receives a “0”. **c** Coding pattern when Bob receives a “0” and Alice receives a “1”. **d** Coding pattern when both Bob and Alice receive a “1”. **e-h** Corresponding electric field distributions generated by the AIM system when applying the coding patterns shown in **a-d**, respectively.

To address potential leakage during transmitting the bits “00”, we introduce an anchor point, which is a predefined and system-recognized safe location in space. This location is either physically occupied by trusted system hardware (e.g., absorptive structures) or electromagnetically shielded, ensuring that no adversary can be present there. When the coding pattern corresponding to “00” is used, the metasurface deliberately focuses the electromagnetic energy toward the anchor point rather than toward Alice or Bob. This design prevents a passive eavesdropper from detecting a strong signal at an unprotected region and deducing the legitimate users’ bits through bit inversion. For the remaining bit combinations (“01”, “10”, and “11”), the electromagnetic energy is precisely focused toward Alice and/or Bob, with negligible radiation elsewhere. The signal energy and bit information received by the legitimate users and the eavesdropper are listed in Supplementary Table II. It can be observed that when the anchor point is employed, the eavesdropper consistently receives very weak energy across all four spatial coding patterns used by the metasurface. In contrast, without the anchor point, switching between different metasurface coding patterns causes significant variations in the electromagnetic energy received by the eavesdropper. As a result, the eavesdropper can easily infer the bits transmitted to the legitimate users through a bit inversion operation, leading to severe key leakage. Overall, the anchor-point mechanism ensures that only trusted spatial regions receive the transmitted energy, effectively eliminating potential signal leakage paths.

**Supplementary Table II. Engergy/Bit information received by the legitimate users and eavesdropper with and without the anchor point**

| Coding pattern | Energy/Bit received by Bob | Energy/Bit received by Alice | **Energy/Bit received by eavesdropper with anchor point** | **Energy/Bit received by eavesdropper w/o anchor point** |
| --- | --- | --- | --- | --- |
| Coding #1 | Low/0 | Low/0 | **Low/0** | **High/1** |
| Coding #2 | High/1 | Low/0 | **Low/0** | **Low/0** |
| Coding #3 | Low/0 | High/1 | **Low/0** | **Low/0** |
| Coding #4 | High/1 | High/1 | **Low/0** | **Low/0** |

**Supplementary Note 7. Bit disagreement rate with the eavesdropper located at different locations**

Bit Disagreement Rate (BDR) is a key metric for assessing the security performance of physical-layer key distribution systems. It represents the proportion of bit mismatches between the key sequences of an eavesdropper (Eve) and the legitimate users (Alice and Bob). In this work, BDR is employed to quantify the confidentiality of the proposed MKD system by evaluating Eve’s ability to infer the legitimate key information. A high BDR value indicates that the key sequences derived by Eve and the legitimate users are largely uncorrelated, demonstrating that the metasurface effectively confines the key-related information within the intended spatial region. Conversely, a low BDR would imply potential information leakage. Therefore, maintaining a consistently high BDR between Eve and the legitimate users serves as a clear indicator of the system’s strong resilience against passive eavesdropping and its capability to achieve secure physical-layer key distribution. Therefore, we evaluated the BDR between the keys generated by the legitimate users and those obtained by an eavesdropper placed at five randomly selected positions. These positions are illustrated in Supplementary Fig. 5. At each position, Eva attempts to reconstruct a key from the EM signals incident at her location, using the same signal processing pipeline as the legitimate users.


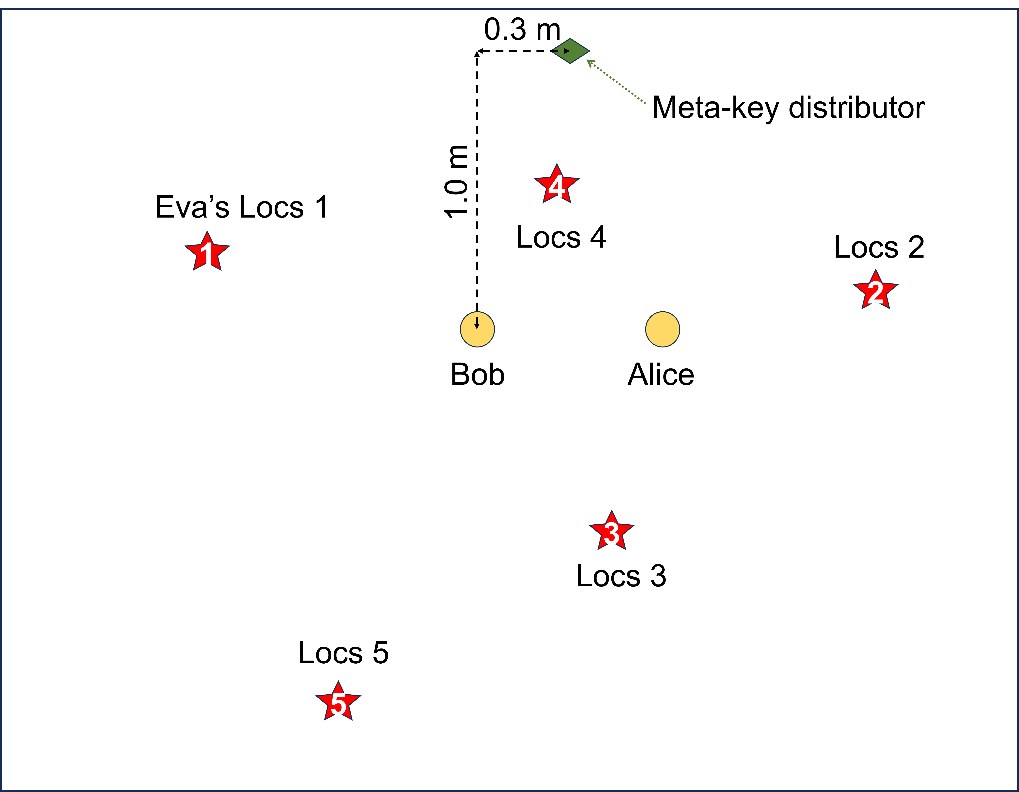


**Supplementary Fig. 5. Top view of the experimental scene layout, showing the randomly selected locations of Eva for meta-key eavesdropping.**

**Supplementary Note 8. Performance comparison with state-of-the-art metasurface-based key generation approaches**

To validate the effectiveness of the proposed system, we compared the MKD system with several representative state-of-the-art metasurface-based key generation approaches in terms of key generation rate (KGR), bit error rate (BER), and security performance (e.g., bit disagreement rate with the eavesdropper). It should be noted that, due to the lack of the specific hardware used in those studies, particularly the programmable metasurfaces, we were unable to reproduce their experimental setups and results within a short period. Therefore, we compared the performance of our proposed method with the reported results in those publications. The comparative results are summarized in Supplementary Table III. It is indicated that the proposed MKD system achieved a competitive KGR while maintaining a low BER, highlighting its effectiveness and potential for secure wireless applications.

**Supplementary Table III.**

**Comparison the with state-of-the-art metasurface-based key generation methods**

|  | **Key generation rate** | **Bit error rate** | **Bit disagreement rate with the eavesdropper** |
| --- | --- | --- | --- |
| **[40]** | 1100.00 bit/s | 0.08 | / |
| **[41]** | 250.00 bit/s | / | 0.48 |
| **[42]** | 30.81 bit/s | / | 0.46 |
| **MKD** | **400 bit/s** | **0.03** | **0.44** |

Note: “/” indicates information that was not reported in the referenced publications.

**References**

[R1]. Tretter, S A. Introduction to Bose Chaudhuri Hocquenghem codes. *Technical Report, the Goddard 1967 Summer Workshop*, 1967.

[R2]. Penard, W, and Van Werkhoven, T. On the secure hash algorithm family. *Cryptography in context*. 1-18 (2008).
